# Determination of *SIRT1* rs12778366, *FGFR2* rs2981582, *STAT3* rs744166, and *RAGE* rs1800625 Single Gene Polymorphisms in Patients with Laryngeal Squamous Cell Carcinoma

**DOI:** 10.1155/2019/3907232

**Published:** 2019-11-12

**Authors:** Virgilijus Uloza, Toma Tamauskaite, Alvita Vilkeviciute, Agne Pasvenskaite, Vykintas Liutkevicius, Rasa Liutkeviciene

**Affiliations:** ^1^Department of Otolaryngology, Academy of Medicine, Lithuanian University of Health Sciences, Kaunas, Lithuania; ^2^Academy of Medicine, Lithuanian University of Health Sciences, Kaunas, Lithuania; ^3^Neuroscience Institute, Lithuanian University of Health Sciences, Kaunas, Lithuania

## Abstract

**Purpose:**

To determine the frequency of the genotype of signal transducer and activator of transcription protein 3 (STAT3) rs744166, sirtuin (SIRT1) rs12778366, fibroblast growth factor (FGFR2) rs2981582, and advanced glycosylation end product-specific receptor (RAGE) rs1800625 gene polymorphisms in patients with laryngeal squamous cell carcinoma (LSCC).

**Methods:**

A total of 944 subjects were evaluated, which includes 144 patients with LSCC and 800 healthy controls. The genotyping of *STAT3* rs744166, *SIRT1* rs12778366, *FGFR2* rs2981582, and *RAGE* rs1800625 was carried out using the RT-PCR.

**Results:**

The analysis of *STAT3* rs744166, *SIRT1* rs12778366, and *FGFR2* rs2981582 gene polymorphisms did not reveal any differences in genotype distribution between the patients with LSCC and the control subjects. However, statistical analysis revealed that genotypes (AA, AG, and GG) of rs1800625 in *RAGE* gene were distributed statistically significantly differently between patients and controls (61.1%, 30.6%, and 23.6% vs. 72.5%, 25.8%, and 1.8%, respectively; *p* < 0.001). Additionally, statistical significance was observed in allele distribution between these two groups, i.e., allele G at rs1800625 was more frequently observed in the patient group than in controls (23.6% vs. 14.6%; *p* < 0.001).

**Conclusion:**

*RAGE* rs1800625 gene polymorphism may play a significant role in laryngeal squamous cell carcinoma development.

## 1. Introduction

The TNM classification for cancers of the head and neck includes tumors of the nasal cavities, paranasal sinuses, oral cavity and larynx, nasopharynx, oropharynx, and hypopharynx [1]. Laryngeal squamous cell carcinoma (LSCC) is one of the largest subgroups, which accounts for 30%-40% of all malignant tumors of the head and neck region [2, 3]. According to the International Agency for Research on Cancer and European Cancer Observatory (ECO), the age-standardized incidence rate of LSCC in Europe is 4.4/100 000 [4]. There were reported 177422 new cases of LSCC and 94771 deaths from LSCC worldwide in 2018 [5]. Despite the improvements in surgical techniques, chemotherapy, and radiotherapy, the 5-year survival rates remain less than 60% [4]. It is well known that LSCC is a complex disease, which is caused by many environmental and genetic factors. The environmental factors that are reported to be associated with the increased risk of LSCC include smoking, alcohol consumption, exposure to carcinogens in the work environment, nutrition, and viral infections with human papilloma virus (HPV) and Epstein-Barr virus (EBV) [6–11]. In the last years, increasing interest has been focused on the role of gene polymorphisms in cancer development and progression [8, 12, 13].

STAT proteins are a family of cytoplasmic transcription factors, which play an important role in the signal transduction through cytokines, hormones, and growth factors. There are evidences that signal transducer and activator of transcription protein 3 (STAT3) is implicated in the development and progression of cancer and plays a role in inducing neoplastic transformation. STAT3 participates in a series of tumorigenic processes such as cell proliferation and survival, antiapoptosis, angiogenesis, immune evasion, and inflammation [14]. Moreover, STAT3 can be activated by a variety of ligands that respond to massive signals such as IL-6, TNF-*α*, and VEGF [15–17]. Aberrant expression and constitutive activation of STAT3 are involved in a broad range of human malignancies, including gastric, breast, prostate, and non-small-cell lung cancers [15, 16, 18–21].

The sirtuins (SIRT) are a highly conserved family of NAD-dependent class III deacetylases that helps to regulate the lifespan of diverse organisms. Mammalian sirtuins consist of 7 members, SIRT1–SIRT7, and some of them, especially SIRT1, have been shown to play relevant roles in the regulation of aging and longevity or in the pathogenesis of age-related metabolic diseases [22–24]. Upregulation of SIRT1 has been reported in breast cancer [25], prostate cancer [26], acute myeloid leukemia [27], and primary colon cancer [28]. It is known that SIRT1 can act both as a tumor promoter and as a suppressor [29, 30]. The apparent opposed roles of SIRT1 seem contradictory, but the multiple functions of SIRT1 made this possible. SIRT1 can negatively regulate both tumor suppressors (p53, FOXO) and oncogenic protein (survivin, *β*-catenin, and NF-*κ*B) multiple pathways. The role of SIRT1 in tumorigenesis might also depend on the temporal and spatial distribution of different SIRT1 upstream regulators and downstream targets [31].

The human fibroblast growth factor (FGF) and its receptor families consist of 22 structurally related FGFs and four high-affinity tyrosine kinase FGF receptors (FGFR1 to 4) that are important for a cell signaling process [32]. The formation of the FGF-FGFR complex activates the intracellular tyrosine kinase, which mediates signal transduction through the direct phosphorylation of adaptor proteins [32, 33]. These complex FGF signaling networks are crucial in the multiple cell biological activities, such as proliferation, differentiation, mitogenesis, migration, and apoptosis, and thus are implicated in tumorigenesis [32–35]. Fibroblast growth factor receptor 2 (FGFR2) belongs to the FGFR family of tyrosine kinase receptors and contributes to the process of tumorigenesis through cell growth, invasiveness, motility, and angiogenesis. It should also be noted that, if the cancer cells overexpress an FGFR and can stimulate the cancer cells, a paracrine loop should be created [32]. At the present a huge interest is focused on FGFR2 polymorphisms, as it may have important implications in breast and other cancer carcinogeneses [36–39].

The receptor for advanced glycosylation end products (RAGE) is referred as a pattern recognition receptor that controls the innate immunity and belongs to the immunoglobulin superfamily of cell surface molecules with a broad spectrum of ligand specificities [40]. These structurally distinct ligands include the prototype of high-mobility group family proteins, members of the S100/calgranulin protein family, extracellular matrix proteins, *β*-amyloid, phosphatidylserine, complement C3a, and some advanced glycation end products [41]. Through interacting with its diverse ligand families, RAGE orchestrates many intracellular signaling pathways to control a variety of cellular processes, such as inflammation, apoptosis, proliferation, and autophagy [41]. Scientists suggest that RAGE plays important roles in several pathophysiologic processes such as cancer [42].

The aim of this study was to determine the possible involvement of *STAT3*, *SIRT1*, *FGFR2*, and *RAGE* gene polymorphisms in LSCC patients as to the best of our knowledge, all these four gene polymorphisms are studied for the first time in LSCC patients.

## 2. Methods and Materials

### 2.1. Ethics Statement

The study was approved by the Ethics Committee for Biomedical Research in Lithuanian University of Health Sciences (LUHS) (permission number is BE-2-34). All subjects provided written informed consent in accordance with the Declaration of Helsinki (the World Medical Association Declaration of Helsinki on Ethical Principles for Medical Research Involving Human Subjects). The study was conducted in the Department of Otolaryngology of LUHS and in the Laboratory of Ophthalmology, Neuroscience Institute of LUHS.

### 2.2. Study Population

A total of 944 subjects were evaluated, which includes 144 patients with LSCC and 800 healthy controls (reference group) (Table 1).

The control group included healthy subjects with no complains related to laryngeal disorders (507 women and 293 men, aged from 19 to 90 years). A voluntary agreement to participate in this research study was obtained from each participant.

The LSCC group consisted of 135 males and 9 females, who underwent surgical treatment at the Department of Otorhinolaryngology and at the Oncological Hospital of LUHS. The age of the LSCC patients ranged from 30 to 86 years (median 63 years). The clinical diagnosis of laryngeal malignancy was based on patients' complaints, typical signs revealed on video laryngoscopy and direct microlaryngoscopy, and the data of neck CT scan or NMR. The pathohistological diagnosis of LSCCs was proved at the Department of Pathology of LUHS.

### 2.3. DNA Extraction, Genotyping, and Statistical Analysis

The methods used in our research were described in previous studies [43, 44].

## 3. Results

The analysis of Hardy-Weinberg equilibrium on 144 patients and 800 healthy subjects revealed that any SNPs did not deviate from Hardy-Weinberg equilibrium (data shown in Table 2).

The analysis of genotype and allele distribution was performed on all 944 subjects (Table 3). Our study results showed that genotypes (AA, AG, and GG) of rs1800625 in RAGE gene were distributed statistically significantly differently between patients and controls (61.1%, 30.6%, and 8.3% vs. 72.5%, 25.8%, and 1.8%, respectively; *p* < 0.001) and allele G at rs1800625 was more frequently observed in the patient group than in controls (23.6% vs. 14.6%; *p* < 0.001) (Table 3).

Binomial logistic regression analysis was performed to evaluate SNPs as the risk factors for LSCC development (Table 4).

Genetic risk models revealed statistically significant variables only in the analysis of rs1800625. Results showed an 8.4-fold increased risk of LSCC development under the codominant (OR = 8.377, 95% CI: 2.880-24.368; *p* < 0.001), a 1.7-fold increased risk under the dominant (OR = 1.677; 95% CI: 1.112-2.529; *p* = 0.014), and a 7.6-fold increased risk under the recessive (OR = 7.623; 95% CI: 2.643-21.989; *p* < 0.001) models as well as under the additive model which shows that each copy of allele G increases the risk of LSCC development by 1.8-fold (OR = 1.844; 95% CI: 1.301-2.613; *p* = 0.001) (Table 4). According to AIC, the best genetic models were codominant and recessive models which show G/G genotype to be associated with an increased risk of LSCC development as well as the additive model which shows G allele association with LSCC development.

## 4. Discussion

We have chosen to investigate four genes SIRT1, FGFR2, STAT3, and RAGE which are known to be closely associated with different types of cancer and are related to pathogenetic processes [45–48].

It has been suggested that a significant increase of SIRT1 expression in hepatocellular carcinoma [23]; breast, prostate, ovarian, gastric, and colon cancers [25, 26, 28, 49, 50]; glioblastoma [51]; and lymphoma [52] might be associated with the development and invasion of tumors.

Few years ago, Noguchi et al. [53] investigated SIRT1 expression to clarify its biological behavior and identify its usefulness as a biomarker for head and neck squamous cell carcinoma (HNSCC). The study showed that 79.6% of STIR1 in HNSCC tissue and nearly all normal tissues were positively stained by immunohistochemical staining of SIRT1 with expression predominated in cases involving patients aged >65 years, lymph node negative, and early clinical stage cases. Thus, this analysis revealed that expression of STIR1 in HNSCC is an independent and good indicator of prognosis.

It was found that Rs12778366 polymorphism of SIRT1 gene is associated with breast cancer [54]. Findings of Rizk et al. revealed that SIRT1 rs12778366 TT genotypes were more frequent in CC and CT genotypes and were associated with histological grade of cancer and lymph node status. SIRT1 rs12778366 TT genotype also correlated with negative estrogen receptor and progesterone receptor statuses. The T allele frequency was higher in breast cancer patients than in normal subjects. However, in the current study, no differences in genotype (TT, TC, and CC) distribution were observed between the control and LSCC groups (80.6 vs. 73.6%, 17.6 vs. 25.0%, and 1.8 vs. 1.4%, respectively; *p* = 0.111).

Tyrosine kinase FGF receptor FGFR2 was found to be overexpressed in bladder [55] and lung cancer [56]. In addition, a study carried out by Zhang et al. [57] revealed that expression of FGFR2 correlated with the occurrence and development of LSCC. Their results showed that expression of FGFR2 from LSCC to para-carcinoma and normal laryngeal mucosa tissues is declined, with statistical significance (*H* = 11.4573, *p* = 0.01). The quantitative expression of FGFR2 protein was relatively higher (FI = 1.8776 ± 0.1683) in LSCC than in para-carcinoma (FI = 1.1815 ± 0.2710) and normal laryngeal mucosa (FI = 1.0100 ± 0.1341) tissues.

The importance of *FGFR2 rs2981582* gene polymorphism was studied in breast [38, 58–66] and prostate cancer [67]. A study in Tunisian population reported that subjects with AA genotype of *FGFR2* rs2981582 had increased risk of breast cancer [68]. Subsequently, Butt et al. [59] in their cohort study in Swedish population confirmed the association between AA genotypes of *FGFR2* rs2981582 and increased breast cancer risk. In contrast, Chen et al. [38] in their study revealed that GA and AA genotypes of *FGFR2* rs2981582 appear to be associated with lower mammographic density and reduced breast cancer risk. Environmental factors and racial/ethnic differences that vary among populations may affect the associations between SNPs and risk of breast cancer. It could be explained by modulating complex interactions between various genes.

FGFR is implicated in the development and progression of the majority of the cancers; therefore, the exact and known mechanisms could be developed to block FGFR activation in cancer cells in the next future [69, 70].

The current study also evaluates the importance of *FGFR2 rs2981582* gene polymorphism in LSCC. All genotypes (GG, GA, and AA) were similar in the control and LSCC groups (42.1 vs. 43.8%, 43.9 vs. 41.7%, and 14.0 vs. 14.6%, respectively; *p* = 0.886). This analysis revealed that there was no statistically significant difference between *FGFR2 rs2981582* gene polymorphism in LSCC and that in the control groups.

Several reports described the influence of STAT3 in the tumor development of colorectal adenocarcinoma, hepatocellular carcinoma, multiple myeloma, glioblastoma, and prostate, head, and neck cancers [71–76]. In 2008, Liu et al. [77] performed a study to investigate the expression and activation of STAT3 in laryngeal carcinoma. The overexpression of STAT3 was determined in all samples of laryngeal squamous cell carcinoma. The mRNA levels of STAT3 were 2.1-fold higher in carcinoma tissue than in control mucosa, respectively. In addition, the protein levels of STAT3 and p-STAT3 were 1.6- and 4.5-fold higher in carcinoma tissue than in control mucosa. It shows that STAT3 is important in the development of LSCCs and represents a potential novel molecular target for therapy to improve survival of patients with LSCC. Several other studies [78, 79] were performed to determine the influence of the Janus-activated kinase (JAK)/STAT inhibitor AG490 on proliferation and apoptosis of Hep-2 human laryngeal cancer cells and to determine whether there was any inhibition by AG490 of the JAK/STAT3 signaling pathway. It was determined that AG490 inhibits significant proliferation, invasion, vasculogenic mimicry, and induced apoptosis of laryngeal carcinoma cells through downregulation of STAT3, suggesting a potential target for LSCC treatment.

A literature search for information on STAT3 rs744166 polymorphism has yielded few studies on gastric, colon, and lung cancer [80, 81, 80, 19]. Yuan et al. [81] showed that rs744166 polymorphism of the STAT3 gene, along with environmental factors, might be associated with the development of gastric cancer. The TC genotype (adjusted OR = 0.60, 95% CI = 0.39-0.92, and *p* = 0.020) and CC genotype (adjusted OR = 0.41, 95% CI = 0.21-0.80, and *p* = 0.009) were associated with a decreased risk of gastric cancer comparing to the TT genotype. In addition, Rocha et al. [80] provided the evidence that *STAT*3 rs744166 G allele is an independent risk factor for gastric cancer. Moreover, Ryan et al. [82] found that rs744166 in *STAT3* was associated with a colon cancer risk, while Jiang et al. [19] determined that carriers of *STAT3* rs744166 have a significantly decreased risk of non-small-cell lung cancer. In the study reported here, association between *STAT3* rs744166 and LSCC risk was not determined. Statistical analysis showed that genotypes (AA, AG, and GG) of rs744166 in *STAT3* gene were not distributed significantly different between patients and controls (32.6%, 47.2%, and 20.1% vs. 35.4%, 45.4%, and 19.2%, respectively; *p* = 0.818). The same statistical significance was observed comparing allele distribution in patients and controls (allele A at rs744166 in the patient group—56.3%, in controls—58.1%; *p* = 0.566).

Ample studies have suggested several *RAGE* gene polymorphisms, alone or in combination with other factors, which are associated with the development or progression of various types of cancer—such as gastric, lung, colorectal, breast, cervical, and ovarian cancers [83–90]. Genetic variations in gene sequence have a potential to alter the function or of *RAGE*, leading to changes in its final bioavailability and, thus, the carcinogenesis [84].

A study conducted by Wang et al. [86] showed that expression of RAGE was reduced in tissues from human lung cancer patients. It should be noted that the polymorphisms of *RAGE*, in particular the −429T/C (rs1800625) and 2184A/G (rs2070600) polymorphisms, were associated with the genesis and progression of lung cancer. The levels of serum sRAGE and tissue RAGE potentially could be an effective and convenient diagnostic biomarker for lung cancer, and the presence of *RAGE* polymorphism may aid the diagnosis of lung cancer and the clinical assessment of prognosis.

Su et al. [84] study showed that *RAGE* rs1800625 is associated with the risk and/or progression of oral squamous cell carcinoma. Moreover, a study revealed that the *RAGE* gene polymorphism rs1800625 not only conferred an increased risk of oral cancer but also was associated with late-stage and large-size tumors. This study also found that individuals who carry at least 1 polymorphic allele of rs1800625, smoke, and chew betel nuts are more susceptible to oral cancer.

Our study was the first to assess the association between *RAGE* rs1800625 and LSCC. It was found that the GG genotype was more frequent in the LSCC group compared with the healthy controls (8.3 vs. 1.8%, respectively; *p* < 0.00) and the AA genotype was less frequent in the LSCC group compared with healthy control group (61.1 vs. 72.5%, respectively; *p* < 0.001). Allele G at rs1800625 was more frequently observed in the patient group than in controls (23.6% vs. 14.6%; *p* < 0.001).

Moreover, *RAGE* rs1800625 analysis revealed that there were significant variables in the codominant (OR = 8.377; 95% CI: 2.880-24.368; *p* < 0.001), recessive (OR = 7.623; 95% CI: 2.643-21.989; *p* < 0.001) and additive (OR = 1.844; 95% CI: 1.301-2.613; *p* = 0.001) models of the patients with LSCC and the control group.

## 5. Conclusions

In conclusion, *RAGE* rs1800625 gene polymorphism might play a significant role in laryngeal squamous cell carcinoma development. To our knowledge, there are no reports on the association of *RAGE* gene polymorphism with LSCC, so its role as a biomarker for prognosis of LSCC development cannot yet be confirmed. For this reason, further studies are needed to explore and confirm this association.

## Figures and Tables

**Table 1:** Demographic characteristics of the study population.

Characteristic	Groups		*p* value
	LSCC *n* = 144	Control *n* = 800	
Male, *n* (%)	135 (93.8)	293 (36.6)	<0.001^∗∗^
Female, *n* (%)	9 (6.2)	507 (63.4)	
Age ± SD Median	62.63 (0.794) 63	50.84 (0.523) 53	<0.001^∗∗^

^∗∗^significant; LSCC: laryngeal squamous cell carcinoma.

**Table 2:** Analysis of allele frequencies and genotype distribution with Hardy-Weinberg equilibrium (HWE).

SNP	Allele frequency		Genotype distribution	HWE *p* value
rs12778366	C (0.11)	T (0.89)	16/177/751	0.143
rs2981582	A (0.36)	G (0.64)	133/411/400	0.100
rs744166	A (0.42)	G (0.58)	183/431/330	0.05
rs1800625	G (0.14)	A (0.86)	16/225/611	0.654

SNP: single-nucleotide polymorphism.

**Table 3:** Frequency of the genotypes of rs12778366, rs2981582, rs744166, and rs1800625 polymorphisms in the LSCC and in the control groups.

Gene marker	Genotype/allele	Control *n* (%) (*n* = 800)	LSCC *n* (%) (*n* = 144)	*p* value
SIRT1 rs12778366	TT	645 (80.6)	106 (73.6)	0.111
	TC	141 (17.6)	36 (25.0)	
	CC	14 (1.8)	2 (1.4)	
	T	1431 (89.4)	248 (86.1)	0.098
	C	169 (10.6)	40 (13.9)	

FGFR2 rs2981582	GG	337 (42.1)	63 (43.8)	0.886
	GA	351 (43.9)	60 (41.7)	
	AA	112 (14.0)	21 (14.6)	
	G	1025 (64.1)	186 (64.6)	0.865
	A	575 (35.9)	102 (35.4)	

STAT3 rs744166	AA	283 (35.4)	47 (32.6)	0.818
	AG	363 (45.4)	68 (47.2)	
	GG	154 (19.2)	29 (20.1)	
	A	929 (58.1)	162 (56.3)	0.566
	G	671 (41.9)	126 (43.7)	

RAGE rs1800625	AA	580 (72.5)	88 (61.1)	**<0.001**
	AG	206 (25.8)	44 (30.6)	
	GG	14 (1.8)	12 (8.3)	
	A	1366 (85.4)	220 (76.4)	**<0.001**
	G	234 (14.6)	68 (23.6)	

HWE: Hardy-Weinberg equilibrium; SIRT1: sirtuin 1 gene; FGFR2: fibroblast growth factor receptor 2 gene; STAT3: signal transducer and activator of transcription 3 gene; RAGE: advanced glycosylation end product receptor gene; LSCC: laryngeal squamous cell carcinoma. *p* values indicated in bold are statistically significant.

**Table 4:** Binomial logistic regression analysis of rs12778366, rs2981582, rs744166, and rs1800625 polymorphisms in the laryngeal squamous cell carcinoma and in the control groups.

Model	Genotype	^∗^aOR (95% CI)	*p*	AIC
SIRT1 rs12778366				
Codominant	**T/C** **C/C**	1.519 (0.951-2.426) 1.307 (0.241-7.095)	0.080 0.757	625.205
Dominant	**T/C+C/C**	1.506 (0.953-2.380)	0.080	623.235
Recessive	**C/C**	1.194 (0.221-6.461)	0.837	626.200
Overdominant	**T/C**	1.513 (0.948-2.413)	0.083	623.298
Additive	**—**	1.421 (0.937-2.155)	0.098	624.571
FGFR2 rs2981582				
Codominant	**G/A** **A/A**	0.951 (0.624-1.452) 0.947 (0.525-1.705)	0.818 0.855	628.177
Dominant	**G/A+A/A**	0.950 (0.641-1.408)	0.799	626.177
Recessive	**A/A**	0.970 (0.559-1.683)	0.914	626.230
Overdominant	**G/A**	0.965 (0.650-1.433)	0.859	626.210
Additive	**—**	0.968 (0.734-1.276)	0.816	626.188
STAT3 rs744166				
Codominant	**A/G** **G/G**	1.216 (0.783-1.887) 1.378 (0.790-2.404)	0.384 0.259	626.801
Dominant	**A/G+G/G**	1.260 (0.835-1.902)	0.271	625.015
Recessive	**G/G**	1.233 (0.752-2.023)	0.406	625.563
Overdominant	**A/G**	1.088 (0.736-1.608)	0.672	626.063
Additive	**—**	1.179 (0.898-1.548)	0.235	624.830
RAGE rs1800625				
Codominant	**A/G** **G/G**	1.371 (0.887-2.120) 8.377 (2.880-24.368)	0.156 **<0.001**	611.143
Dominant	**A/G+G/G**	1.677 (1.112-2.529)	**0.014**	620.248
Recessive	**G/G**	7.623 (2.643-21.989)	**<0.001**	611.128
Overdominant	**A/G**	1.223 (0.796-1.877)	0.358	625.406
Additive	**—**	1.844 (1.301-2.613)	**0.001**	614.547

^∗^aOR: adjusted odds ratio by age and gender; CI: confidence interval; AIC: Akaike Information Criterion.

## Data Availability

The data used to support the findings of this study are available from the corresponding author upon request.
